# The Impact of Homogenization Techniques and Conditions on Water‐In‐Oil Emulsions for Casein Hydrolysate–Loaded Double Emulsions: A Comparative Study

**DOI:** 10.1002/fsn3.4525

**Published:** 2024-10-18

**Authors:** Pelin Salum, Çağla Ulubaş, Onur Güven, Mustafa Cam, Levent Yurdaer Aydemir, Zafer Erbay

**Affiliations:** ^1^ Department of Food Engineering, Faculty of Engineering Adana Alparslan Turkes Science and Technology University Adana Turkey; ^2^ Department of Mining Engineering, Faculty of Engineering Adana Alparslan Turkes Science and Technology University Adana Turkey; ^3^ Department of Food Engineering, Faculty of Engineering Erciyes University Kayseri Turkey

**Keywords:** double emulsion, high‐pressure, homogenization, rotor–stator, ultrasound, water‐in‐oil emulsion

## Abstract

This study aims to evaluate homogenization techniques and conditions for producing stable, small droplet‐size water‐in‐oil (W/O) emulsions intended for incorporation into casein hydrolysate–loaded double emulsions. Three commonly used homogenization methods; rotor–stator, ultrasonic, and high‐pressure homogenization were individually optimized utilizing response surface methodology. Instances of over‐processing were observed, particularly with the rotor–stator and ultrasonic homogenizers under specific conditions. Nevertheless, optimal conditions were identified for each technique: 530 s at 17,800 rpm agitation speed for the rotor–stator homogenizer, 139 s at 39% amplitude for the ultrasonic homogenizer, and 520 s at 1475 bar for the high‐pressure homogenizer. Subsequently, the W/O emulsions produced under optimal conditions and their respective W_1_/O/W_2_ double emulsions were compared. The rotor–stator and high‐pressure homogenized W/O emulsions exhibited comparable narrow droplet‐size distributions, as indicated by similar Span values. However, high‐pressure homogenization failed to sufficiently minimize droplet size. Ultrasonic homogenization resulted in droplets at the 1‐μm scale but yielded more polydisperse droplet‐size distribution. According to TOPSIS analysis, an emulsion with a viscosity of 93.1 cP (centiPoise), a stability index of 93.8%, a D(90) of 0.67 μm (0th day), and a D(90) of 0.75 μm (30th day) produced using a rotor–stator was selected. Additionally, double emulsions containing primary emulsions prepared with the rotor–stator method demonstrated higher viscosity, narrower droplet‐size distribution, and lower creaming compared to other samples. This investigation sheds light on the influence of homogenization techniques on emulsion properties, providing valuable insights for optimizing double emulsion formulations.

## Graphical abstract

This study optimizes homogenization techniques for water‐in‐oil (W/O) emulsions in double emulsions. Rotor–stator, ultrasonic, and high‐pressure homogenization methods were optimized based on emulsions' viscosity, stability, and droplet size in both W/O and double emulsions. This research offers insights for refining double emulsion formulations by considering the impact of homogenization techniques on emulsion properties.

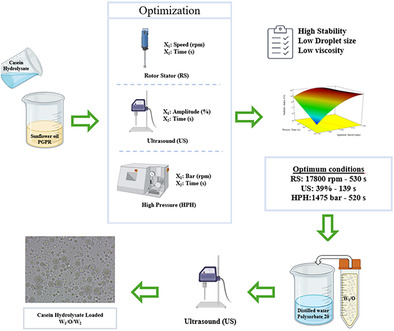

## 1 Introduction

Hydrolysis of casein produces bioactive peptides that can positively impact various physiological systems, including cardiovascular, digestive, endocrine, immune, and nervous systems. However, it is essential for these peptides to remain active during the digestion (Korhonen 2002) and commercial production of peptide‐rich food additives faces challenges (Giroldi et al. 2021). Encapsulation is a promising solution to address these issues (Aguilar‐Toala et al. 2022). Utilizing water‐in‐oil‐in‐water (W_1_/O/W_2_) double emulsions for peptide and amino acid encapsulation provides benefits, such as simple processing, easy control of process parameters, and cost‐effective production (Giroldi et al. 2021; Ye, Kim, and Park 2010). However, the preparation of W_1_/O/W_2_ emulsions is more complex than conventional emulsions. In addition, instability in long‐term storage is the main problem in food‐related applications of W_1_/O/W_2_ emulsions (Muschiolik and Dickinson 2017; Heidari et al. 2022). The stability of double emulsions depends on both the composition of the emulsions and the emulsification conditions employed. Although the stability can be enhanced by modifying the structure of the outer water phase and increasing its viscosity, the instability of double emulsions is strongly caused by the instability of the primary W/O emulsions (Muschiolik and Dickinson 2017).

A two‐stage emulsification technique, in which two individual emulsification steps are consequently included as W_1_/O and (W_1_/O)/W_2_ formation, is commonly used to prepare W_1_/O/W_2_ emulsions (Klojdová, Štětina, and Horáčková 2019). In this technique, the properties of the primary emulsion (W/O) play important roles in the production of stable W_1_/O/W_2_ emulsions (Chevalier, Gomes, and Cunha 2021). In addition, the peptides can adversely affect the stability of W/O emulsions due to their interfacial activity, which can disrupt the lipophilic emulsifier at the water/oil interface during emulsification (Jo, Karbstein, and Van Der Schaaf 2019). Therefore, it is necessary to enhance the stability of the W/O emulsion for the encapsulation of casein hydrolysate. In W/O emulsions, steric forces stabilize only the emulsion due to the low electrical conductivity (EC) of the continuous phase. Moreover, the high mobility of water droplets is a cause for sedimentation of, flocculation of, or coalesced W/O emulsions (Ushikubo and Cunha 2014) leading to the low stability of W/O emulsions. Therefore, one approach for improving the stability of double emulsions is to design a W/O emulsion with enhanced stability (Chevalier, Gomes, and Cunha 2021). The emulsification process is one of the most important factors that directly affect the emulsion properties and is most commonly carried out with the input of mechanical energy to the system for the creation of droplet breakup to produce smaller droplets (Lee et al. 2013).

For this reason, high‐energy devices, such as high shear mixers, high‐pressure homogenizers, colloid mills, and ultrasonic homogenizers, are generally used for the production of primary emulsion (W/O) (Kumar et al. 2022; McClements 2016). However, there is no consensus in the literature regarding which method is the most effective one for the preparation of primary emulsions, as each technique has its own set of advantages and disadvantages (McClements 2016; Taha et al. 2020). Rotor–stator (RS)–type conventional homogenizers have been widely used for the preparation of the W/O in the initial stage of W_1_/O/W_2_ production (Muschiolik and Dickinson 2017). Rotor–stator systems create a high shear force between a rotor (a rotating disk) and a stator (a static disk), and turbulence is the primary cause of fluid degradation leading to the formation of smaller droplets. In addition, the rapid rotation of the rotor creates shear stress in the cavity, causing the larger droplets to split into smaller droplets and creation of centrifugal force, which causes the fluid to move from the center of the disks to its periphery (McClements 2016). Another common homogenizer is the high‐pressure homogenizer (HP), composed of a high‐pressure pump, which can apply pressures ranging from 3 to 500 MPa to fluid systems, and a homogenizing valve (Taha et al. 2020). The pump draws the fluid into the chamber and then, forces it through a narrow valve at the end of the chamber. As the fluid system passes through the valve, it is subjected to a combination of intense disruptive forces as a result of sudden decrease in pressure and increase in velocity that cause larger droplets to break up into smaller ones (McClements 2016). The emulsification via high‐pressure homogenization is caused by the effects of turbulence and cavitation, resulting in high‐energy dissipation (van der Graaf, Schroën, and Boom 2005). Over and above these, ultrasonic homogenizers (USs) (sonicators) have been used to produce emulsions. These types of homogenizers use high‐intensity ultrasonic waves to create strong shear and pressure gradients within a material, which disrupt droplets primarily through cavitation and turbulent effects (McClements 2016; Taha et al. 2020). The efficiency of cavitation during sonication is affected by various factors, such as ultrasound frequency, power, amplitude, process temperature, and surface tension or viscosity of the product.

Altuntas, Sumnu, and Sahin (2017) determined that the rotor–stator produced more stable primary emulsions with the smallest droplet sizes compared to ultrasound and microfluidizer. Yildirim, Sumnu, and Sahin (2017) found no significant difference in encapsulation efficiency between the use of rotor–stator and ultrasound, but noted that rotor–stator resulted in smaller droplet sizes. Raviadaran et al. (2019) found similar properties for emulsion produced by ultrasound and microfluidizer, but ultrasound was a more energy‐efficient technique.

The objective of this study was to produce W_1_/O emulsions for use as primary emulsions in W_1_/O/W_2_ systems for the encapsulation of casein hydrolysate. It is well established that emulsion stability is enhanced by reducing droplet size and increasing viscosity (Muschiolik and Dickinson 2017). In general, reducing droplet size also leads to increased viscosity (Pal 1996). However, it becomes challenging for the primary emulsions with high viscosity to disperse effectively in the outer water phase during the secondary emulsification process in the production of W_1_/O/W_2_ emulsions. This contrast highlights the need for the optimization of the emulsification process and the production of a stable W/O emulsion with both low viscosity and small droplet size, which were aimed in this study. The emulsification techniques, which are commonly used in food emulsion formation, such as rotor–stator, high‐pressure, and ultrasonic homogenizers in the preparation of primary emulsions were investigated and individually optimized. The optimization factors were agitation speed and processing time for the rotor–stator, amplitude and processing time for the ultrasonic homogenizer, and pressure and processing time for the high‐pressure homogenizer. The effects of process parameters on the properties of casein hydrolysate–loaded W/O emulsions were analyzed. The emulsification processes were individually optimized, and emulsions produced under optimized process conditions were subsequently compared for the first time according to the authors' knowledge. Additionally, W/O emulsions produced at each optimum condition were utilized as primary emulsions in the preparation of double emulsions and their effects on double emulsion properties were examined.

## 2 Materials and Methods

### 2.1 Materials

The sunflower oil was acquired from a local store, while polyglycerol polyricinoleate (PGPR 4150) and polysorbate 20 (Crillet 1) were obtained as gift samples kindly provided by Palsgaard (Juelsminde, Denmark) and Croda Chemicals (Snaith, UK), respectively. The casein hydrolysate, prepared as outlined in Salum, Ulubaş, et al. (2023), was used as the dispersed phase in the primary emulsions.

### 2.2 Emulsion Production

#### 2.2.1 Preparation of W/O Emulsions

In our previous study, a W/O emulsion with a dispersed phase of 25% (w/w) and an emulsifier concentration of 5% PGPR 4150 (w/w oil) was identified as an appropriate formulation, demonstrating stability and low viscosity (Salum, Ulubaş, et al. 2023). Initially, the specified amount of PGPR and sunflower oil was combined, ensuring the emulsifier's dissolution. Following this, the appropriate quantity of casein hydrolysate was introduced into the oil phase. While the casein hydolysate was prepared in phosphate buffer (0.1 M salt‐free buffer containing sodium dihydrogen phosphate (NaH_2_PO_4_) and disodium hydrogen phosphate (Na_2_HPO_4_) adjusted to pH 8.0), its protein and total solid contents were 3.75% and 4.30%, respectively. Subsequently, the mixture was homogenized separately using a rotor–stator (Ultra‐Turrax, IKA, T‐18, Staufen, Germany), an ultrasonic homogenizer (Bandelin, Sonopuls HD 4200 with TS 103 probe, Berlin, Germany), and a two‐stage high‐pressure homogenizer (APV Lab2000, SPX Flow, Søborg, Denmark), following the experimental design outlined in Section 2.4.1. The production of emulsions was carried out in batches of 50, 30, and 100 g using the rotor–stator (RS), ultrasonic homogenizer (US), and high‐pressure homogenizer (HP), respectively. The quantity of emulsion produced for each method was determined based on preliminary trials, taking into consideration the homogenizer characteristics and capacity.

#### 2.2.2 Preparation of W_1_ /O/W_2_ Emulsions

Double emulsions were produced using primary emulsions prepared at each optimal condition. The formulation of the external water phase and homogenization conditions were used from the optimum parameters determined by Salum et al. (2024). In this context, 30% of the primary emulsion was dispersed in the outer water phase containing 1% Crillet 1. For the second homogenization, an ultrasonic homogenizer (Bandelin, Sonopuls HD 4200 with TS 103 probe, Berlin, Germany) was utilized due to its ability to provide rapid homogenization in a short period. The homogenization was carried out at 54% amplitude for 62 s.

### 2.3 Analysis of Flow Characteristics, Stability, and Average Droplet Sizes of the Emulsions

The present study encompassed the analysis of flow characteristics, stability, and average droplet sizes of the emulsions generated. Furthermore, the temperatures and electrical conductivity of all emulsions were measured immediately after their preparation with a pH/mV/EC/TDS/NaCl/Temp Bench Meter (MW180 MAX, Milwaukee Instruments, USA).

#### 2.3.1 Determination of Flow Behaviors

The flow behaviors of the emulsions were ascertained through a viscometer (DV‐II+ Pro Viscometer, Brookfield Engineering, Middleborough, MA, USA), equipped with a small sample adapter (SSA‐13RD, Brookfield Engineering, Middleborough, MA, USA). The emulsion temperature was adjusted by a water circulation system (ICC Basic Eco 8, IKA, Staufen, Germany) at 35°C. The shear stress values were determined employing the controlled rate ramp method. Subsequently, the dimensionless flow behavior indices (*n'*s) and the consistency coefficients (*K'*s) were calculated using the power‐law model, as described in Equation (1):

(1) $$\tau =K{\left(\dot{\gamma }\right)}^{n}$$

Here, *τ* (Pa) is the shear stress, *γ̇* (1/s) is the shear rate, *K* (Pa.s^n^) is the consistency coefficient, and *n* is the dimensionless flow behavior index.

#### 2.3.2 Determination of Stability Index

The stability index value of the W/O emulsions was assessed by adapting the method outlined by Azarikia et al. (2017). Immediately after the preparation of emulsion, 7 mL of emulsion was transferred into a 15‐mL glass test tube with a flat bottom and then stored in a dark environment at room temperature for 30 days. Photographic documentation of each tube was captured on both the 0th and 30th days of storage using a mobile phone camera. Each tube was positioned within a dark enclosure and illuminated from behind by a 7 W light‐emitting diode (LED) light to enhance internal phase separation visibility and contrast. The height measurements of the emulsion, as well as the separated oil and water phases, were quantified using the ImageJ/Fiji software (ver.1.53c.). Calibration was established using a ruler placed at a fixed position.

(2) $$\text{Stability Index}\;\left(\%\right)≔\left[\left(h_{t}-\left(h_{o}+h_{w}\right)\right)/h_{t}\;\right]\times 100$$

Here, *h*_t_ is the total height, *h*_o_ is the height of the separated oil phase, and h_w_ is the height of the water phase.

#### 2.3.3 Determination of Droplet‐Size Distribution

Two different methods were employed to determine the droplet sizes of emulsions. The droplet sizes of primary W/O emulsions were determined using a microscopy‐assisted digital image analysis technique as detailed by Salum et al. (2022), while the droplet sizes of double emulsions were measured with a laser diffraction particle size analyzer.

The droplet size of primary emulsions (W_1_/O) was determined on the 0th and 30th days. Micrographs were captured using a compound microscope (M83EZ, OMAX Microscopes, Kent, WA, USA) equipped with a 100×/1.25 oil, 160/0.17 objective lens and a 5‐Megapixel CMOS camera (A3550U, OMAX Microscopes, Kent, WA, USA). A minimum of 120 photographs were captured from five distinct microscope slides for each sample. Subsequent image segmentation and droplet‐size analysis were carried out using the Trainable Weka Segmentation (TWS) classifier within the TWS plugin (version 3.2.35) and ImageJ/Fiji software (ver.1.53c.). A comprehensive analysis of over 8000 droplets was carried out for each sample.

Droplet sizes of the W_1_/O/W_2_ emulsions were determined using the Mastersizer 3000 (Malvern Instruments Ltd., Worcestershire, UK) on the 0th and 8th days of storage. The refractive index values of sunflower oil and distilled water were adjusted to 1.472 and 1.333, respectively. Obscuration values were adjusted between 10% and 14%. The measurements were done in triplicate at room temperature and a mixing speed of 2100 rpm was applied during the measurement (Salum, Berktas, Kendirci, et al. 2023).

Based on the droplet‐size data, values for D(90), D[3,2], and D[4,3] were calculated, which, respectively, denote the equivalent volume diameters at 90%, as well as the area‐ and volume‐weighted mean diameters. Moreover, Span value was computed as a measure of droplet‐size distribution width.

#### 2.3.4 Determination of the Retention Efficiency of the Internal Phase of W_1_ /O/W_2_

Retention efficiency was determined using the method described by Salum et al. (2024). In systems where the outer and inner phases have different electrical conductivity (EC) values, the EC measurement can be a useful and straightforward method to assess the retention of the W_1_ phase in W_1_/O emulsions. The EC value of the external phase will increase as the primary emulsion breaks down during the second homogenization process or as the internal phase diffuses into the external phase (Kim et al. 2003; Paula et al. 2018). The EC value will reach its maximum when all the inner phase has moved to the external phase. This represents the theoretical situation where retention, or encapsulation efficiency, is 0.

In this study, the EC values of the outer water phase (W_2_, 1% polysorbate 20 in distilled water) were much lower than those of the inner water phase (W_1_) consisting of casein hydrolysate in a buffer solution. To determine the maximum potential value of EC, a sample was prepared by adding the entire formulation of the internal phase into the external phase without emulsifying process and EC of the samples was measured (1944 μS/cm). Additionally, the EC of the double emulsions was measured immediately after emulsion preparation and on the 8th day of storage. The retention efficiency of the internal phase was calculated as a percentage using the following formula:

(3) $$\text{The retention efficiency of the internal phase}\;\left(\%\right)=\frac{\left({\mathrm{EC}}_{\max,\text{release}}-{\mathrm{EC}}_{\text{emulsion}}\right)}{{\mathrm{EC}}_{\max,\text{release}}}\times 100$$

EC_max,release_ refers to the electrical conductivity value under maximum release conditions (μS/cm) and EC_emulsion_ refers to the electrical conductivity of the double emulsions (μS/cm).

### 2.4 Experimental Design and Statistical Analysis

#### 2.4.1 Experimental Design for Optimization

Response surface methodology was employed to optimize the homogenization conditions for each homogenizer. To this end, experimental conditions were designed using a two‐factor Central Composite Rotatable Design (CCRD) composed of 13 experimental points, as guided by Design Expert Ver. 7.0.0 software. The design comprised five central points and four axial points (*λ* = 1.414), in addition to a two‐level full factorial design for two factors. The experimental CCRDs utilized for the optimization of W/O emulsion preparation using the RS, US, and HP are provided in Table 1.

**TABLE 1:** Experimental designs used in the optimization of the emulsification of water‐in‐oil (W/O) emulsions with different homogenizers.

	Rotor–stator		Ultrasonic homogenizer		High‐pressure homogenizer	
No.	Agitation speed (*x*_1_, rpm)	Time (*x*_2_, s)	Amplitude (*x*_1_, %)	Time (*x*_2_, s)	Pressure (*x*_1_, bar)	Time (*x*_2_, s)
1	17,800 (+1)	530 (+1)	50 (0)	330 (0)	1000 (0)	60 (−1.41)
2	12,500 (0)	360 (0)	39 (−1)	140 (−1)	1000 (0)	330 (0)
3	20,000 (+1.41)	360 (0)	50 (0)	330 (0)	1000 (0)	330 (0)
4	12,500 (0)	360 (0)	35 (−1.41)	330 (0)	1495 (+1)	520 (+1)
5	17,800 (+1)	190 (−1)	50 (0)	60 (−1.41)	1700 (+1.41)	330 (0)
6	12,500 (0)	360 (0)	50 (0)	330 (0)	1000 (0)	600 (+1.41)
7	12,500 (0)	360 (0)	50 (0)	330 (0)	1000 (0)	330 (0)
8	12,500 (0)	600 (+1.41)	50 (0)	330 (0)	505 (−1)	520 (+1)
9	7200 (−1)	190 (−1)	50 (0)	600 (+1.41)	1000 (0)	330 (0)
10	12,500 (0)	360 (0)	61 (+1)	520 (+1)	1000 (0)	330 (0)
11	5000 (−1.41)	360 (0)	39 (−1)	520 (+1)	1495 (+1)	140 (−1)
12	7200 (−1)	530 (+1)	65 (+1.41)	330 (0)	300 (−1.41)	330 (0)
13	12,500 (0)	120 (−1.41)	61 (+1)	140 (−1)	505 (−1)	140 (−1)

In this study, two primary process variables were identified as optimization factors for each homogenizer, with their levels determined through preliminary experiments considering the operational limits of the homogenizers. Specifically, for the rotor–stator, the optimization factors were agitation speed (ranging from 5000 to 20,000 rpm) and processing time (ranging from 120 to 600 s). Regarding the ultrasonic homogenizer, the optimization factors were amplitude (ranging from 35% to 65%) and processing time (ranging from 60 to 600 s). For the two‐stage high‐pressure homogenizer, the optimization factors were pressure (ranging from 300 to 1700 bars for the first stage of homogenization, which was 5 times higher than during the second stage) and processing time (ranging from 60 to 600 s).

#### 2.4.2 Model Fitting for Optimization

The optimization responses, specifically viscosity, stability index, and D(90) values (both at emulsion preparation and after 30 days), were selected to achieve an emulsion characterized by the lowest viscosity and highest stability. Multiple regression analysis was conducted, leading to the derivation of quadratic model equations for each response. In order to verify assumptions regarding the significance of differences in the models related to the linear, quadratic, and interaction effects of each factor, *F‐*statistics were employed for analysis of variance (ANOVA). Insignificant effects (*p* > 0.1) were removed from the models without damaging model hierarchy.

Subsequently, regression coefficients were computed for each finalized model. To ensure the adequacy of the final models, several metrics were checked, including the regression coefficient (*R* ^2^), adjusted regression coefficient (adj‐*R* ^2^), predicted regression coefficient (pre‐*R* ^2^), coefficient of variance (C.V.), adequate precision (Adeq.Precision), and the predicted residual error sum of squares (PRESS) (Myers and Montgomery 2002). Finally, response surfaces were plotted based on the fitted models to visualize the relationships and interactions between optimization factors.

#### 2.4.3 Optimization and Verification

Given the optimization objective of achieving emulsions with the lowest viscosity and highest stability, the responses were defined as the viscosity, stability index, and D(90) values immediately after emulsion preparation and following 30 days of storage. For this purpose, the stability index values were maximized, while other factors were minimized. Equal importance weights (*r* = 3) were assigned to each response. To determine the optimal process conditions for each homogenizer, the desirability function method was employed (Erbay and Icier 2009). In order to validate the estimated theoretical optimum conditions, three emulsion productions were conducted at these conditions for each homogenizer. The estimated response values were then compared with the corresponding experimental values using one sample *t‐*test.

#### 2.4.4 Comparison and Multi‐Criteria Decision Analysis

The comparison of the emulsification techniques was carried out using the emulsions produced under optimum process conditions for each homogenizer. Data analysis for the comparison was performed through ANOVA and the Duncan Post Hoc Test using the SPSS statistical software (Version 22, Chicago, IL, USA), with statistical significance set at *p* < 0.05.

The selection of the most suitable technique was performed using a multi‐criteria decision analysis method known as the Technique for Order Preference by Similarity to Ideal Solution (TOPSIS). In this methodology, the selected alternative should exhibit the greatest geometric distance from the negative ideal solution while maintaining the shortest geometric distance from the positive ideal solution (Hwang and Yoon 1981).

During the determination of these positive and negative ideal solutions, an initial matrix is formed using experimental outcomes, followed by the normalization and weighting of the solution matrix. Essentially, the experimental results establish the parameters for defining the ideal solutions (Himmetagaoglu, Erbay, and Cam 2018). The result of the TOPSIS method is quantified using the concept of “relative closeness (C)” to the positive ideal solution. This numerical value falls within the range of 0 to 1, with a value closer to 1 being indicative of a more favorable solution. In this study, the TOPSIS was applied using the optimization responses with equal weights.

## 3 Results and Discussion

### 3.1 Model Fitting and Effects of Homogenization Parameters

The process parameters of the RS, US, and HP were individually investigated based on the experimental design shown in Table 1. For this purpose, flow behavior parameters, electrical conductivity measurements, droplet‐size distribution data (at the 0th and 30th days of storage), and stability index values of a total of 39 emulsions were evaluated (Tables S1–S3).

All the gathered data were assessed through multiple regression analysis to establish well‐fitting models, and ANOVA was conducted to ensure the validity of these models. The experimental D(90) values of the emulsion produced via RS were subjected to inverse square root transformation to improve the adequacy of the models. The selection of optimization responses was based on properties that exhibited suitable models across all homogenization techniques, facilitating their comparison. Subsequent to refining the models for enhanced adequacy, the ANOVA evaluation for each response variable and the coefficients of the predicted models is presented in Table 2.

**TABLE 2:** Analysis of variance (ANOVA) evaluation for each response variable and the coefficients of the prediction models after removing the insignificant terms from the models.*

Unit	Source*	Viscosity		*D*(90) / 0th day		Stability index		*D*(90) / 30th day	
		*p*	Coefficient	*p*	Coefficient	*p*	Coefficient	*p*	Coefficient
Rotor–stator homogenizer	Model	< 0.0001	100.03	0.0002	0.91	0.0001	92.40	0.0004	0.96
	*x*_1_	< 0.0001	14.71	< 0.0001	0.30	0.0001	9.62	< 0.0001	0.34
	*x*_2_	0.0103	6.55	0.0155	0.10	0.0067	5.08	0.0058	0.15
	*x*_1_ *x*_2_	0.0015	−13.16	0.0626	−0.10	0.0053	−7.51	0.0916	−0.10
	*x*_1_^2^	0.0002	−14.03	0.0623	−0.078	0.0006	−8.16	0.0654	−0.088
	*x*_2_^2^	—	—	—	—	—	—	0.0371	−0.10
	Lack of Fit	0.2600		0.1799		0.1424		0.3087	
	*R* ^2^	0.9439		0.9240		0.9290		0.9414	
	Adj‐*R* ^2^	0.9159		0.8860		0.8935		0.8995	
	Pred‐*R* ^2^	0.7839		0.7367		0.7169		0.7274	
	Adeq Precision	16.858		14.375		15.278		15.156	
	PRESS	952.6		0.25		500.80		0.37	
	C.V.	6.08		11.06		4.54		12.65	
Ultrasonic homogenizer	Model	0.0002	101.85	< 0.0001	5.07	< 0.0001	84.55	0.0001	12.81
	*x*_1_	0.0073	−1.42	0.0001	1.12	—	—	0.0002	2.07
	*x*_2_	0.0017	1.86	0.0003	1.04	< 0.0001	−3.02	0.0136	0.94
	*x*_1_ *x*_2_	0.0490	−1.28	—	—	—	—	0.0003	−2.66
	*x*_1_^2^	—	—	—	—	—	—	0.0007	−1.75
	*x*_2_^2^	0.0001	−3.02	0.0002	1.17	0.0007	1.72	0.0533	0.72
	Lack of Fit	0.4044		0.6329		0.2300		0.7767	
	*R* ^2^	0.9173		0.9246		0.9134		0.9560	
	Adj‐*R* ^2^	0.8759		0.8995		0.8961		0.9246	
	Pred‐*R* ^2^	0.7609		0.8333		0.7867		0.8787	
	Adeq Precision	15.053		19.059		19.872		17.724	
	PRESS	30.30		5.19		21.95		12.86	
	C.V.	1.14		8.82		1.10		6.73	
High‐presure homogenizer	Model	0.0001	94.74	< 0.0001	7.12	0.0004	85.22	< 0.0001	13.37
	*x*_1_	< 0.0001	−3.86	0.0004	−1.72	0.0195	0.85	< 0.0001	−3.19
	*x*_2_	0.0002	−3.56	0.0002	−1.97	0.0010	1.48	0.0118	−0.32
	*x*_1_ *x*_2_	0.0312	1.96	0.0069	−1.52	0.0011	2.05	< 0.0001	−2.25
	*x*_1_^2^	0.0997	1.05	0.0018	1.45	0.0026	1.34	—	—
	*x*_2_^2^	—	—	—	—	—	—	< 0.0001	−0.89
	Lack of Fit	0.6944		0.5583		0.7346		0.6908	
	*R* ^2^	0.9313		0.9329		0.9056		0.9943	
	Adj‐*R* ^2^	0.8969		0.8994		0.8584		0.9915	
	Pred‐*R* ^2^	0.8070		0.7737		0.7529		0.9867	
	Adeq Precision	16.620		17.418		15.186		64.770	
	PRESS	50.30		19.02		14.34		1.45	
	C.V.	1.57		10.48		0.96		2.16	

* While *x*_1_, respectively, represent agitation speed, amplitude, and pressure for rotor‐stator, ultrasonic and high‐pressure homogenizers, *x*_2_ represent processing time for all homogenizers.

Our findings demonstrated that all the models represented highly the variation of experimental data, with *R* ^2^ values exceeding 0.90. Moreover, the differences between *R* ^2^ and Adj‐*R* ^2^ were all below 0.050 (the highest difference was 0.047 in the present models), indicating the absence of statistically insignificant terms in the models (Table 2). Additionally, the discrepancies between Adj‐*R* ^2^ and Pred‐*R* ^2^ values remained below 0.200 (particularly below 0.177 in our models), which signified the models' capacity to accurately predict new observations. Furthermore, all “*Lack of fit*” values exceeded 0.100 with the lowest value being 0.142 and Adeq.Precision values exceeded 4 with the lowest value recorded as 14.375, indicating that the constructed models possessed statistical suitability for estimation (Salum, Berktas, Bas, et al. 2023). Based on these results, the established models were statistically viable for predicting the responses.

**FIGURE 1:**
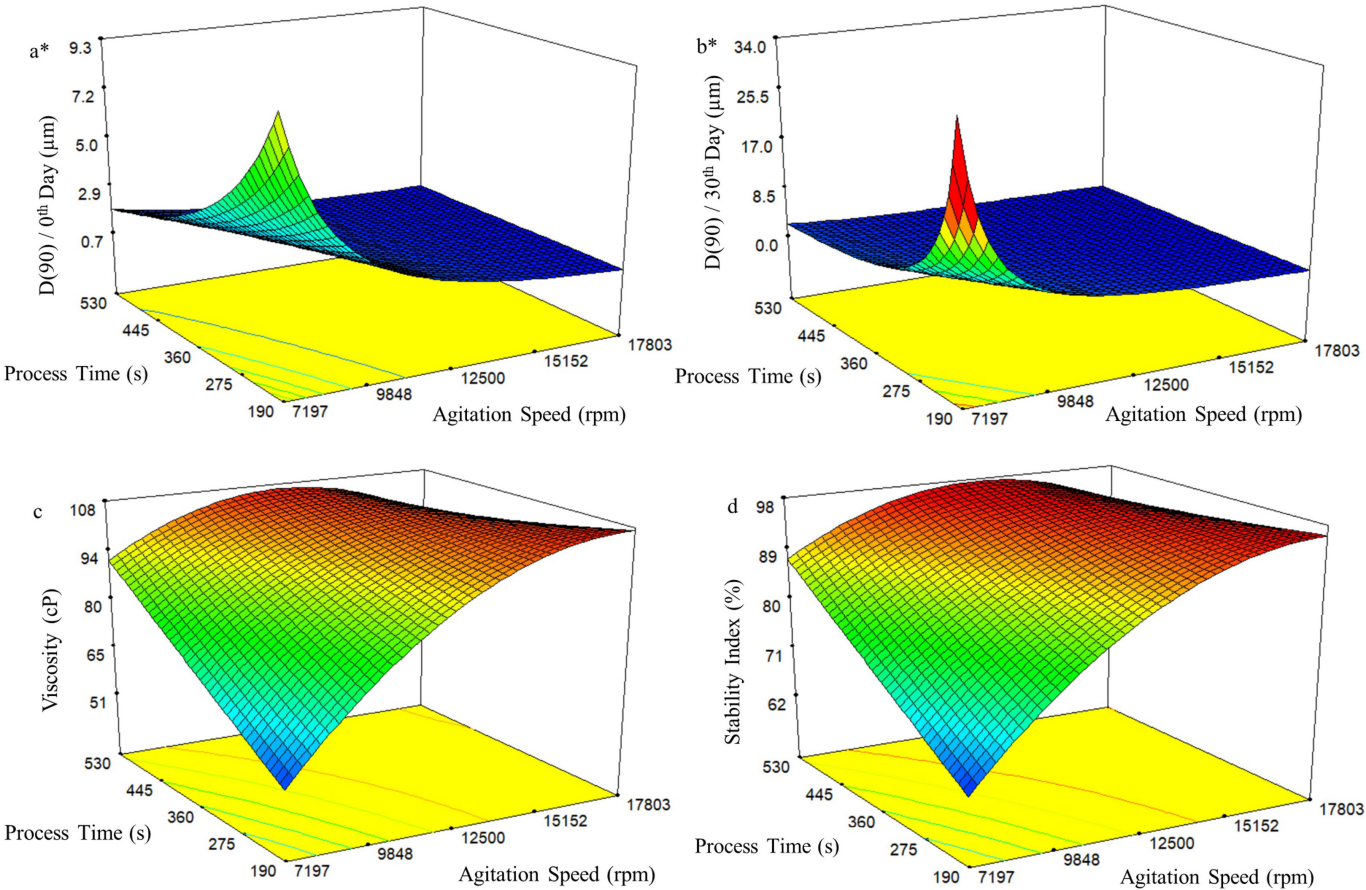
Response surface and contour plots for (a) D(90) at 0th day of storage, (b) D(90) at 30th day of storage, (c) viscosity, and (d) stability index of water‐in‐oil (W/O) emulsions produced with rotor–stator (*transformation).

**FIGURE 2:**
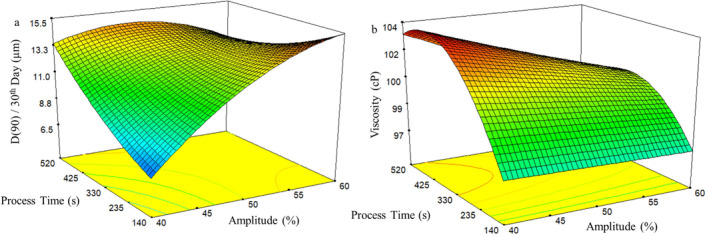
Response surface and contour plots for (a) D(90) at 30th day of storage and (b) viscosity of water‐in‐oil (W/O) emulsions produced with ultrasonic homogenizer.

**FIGURE 3:**
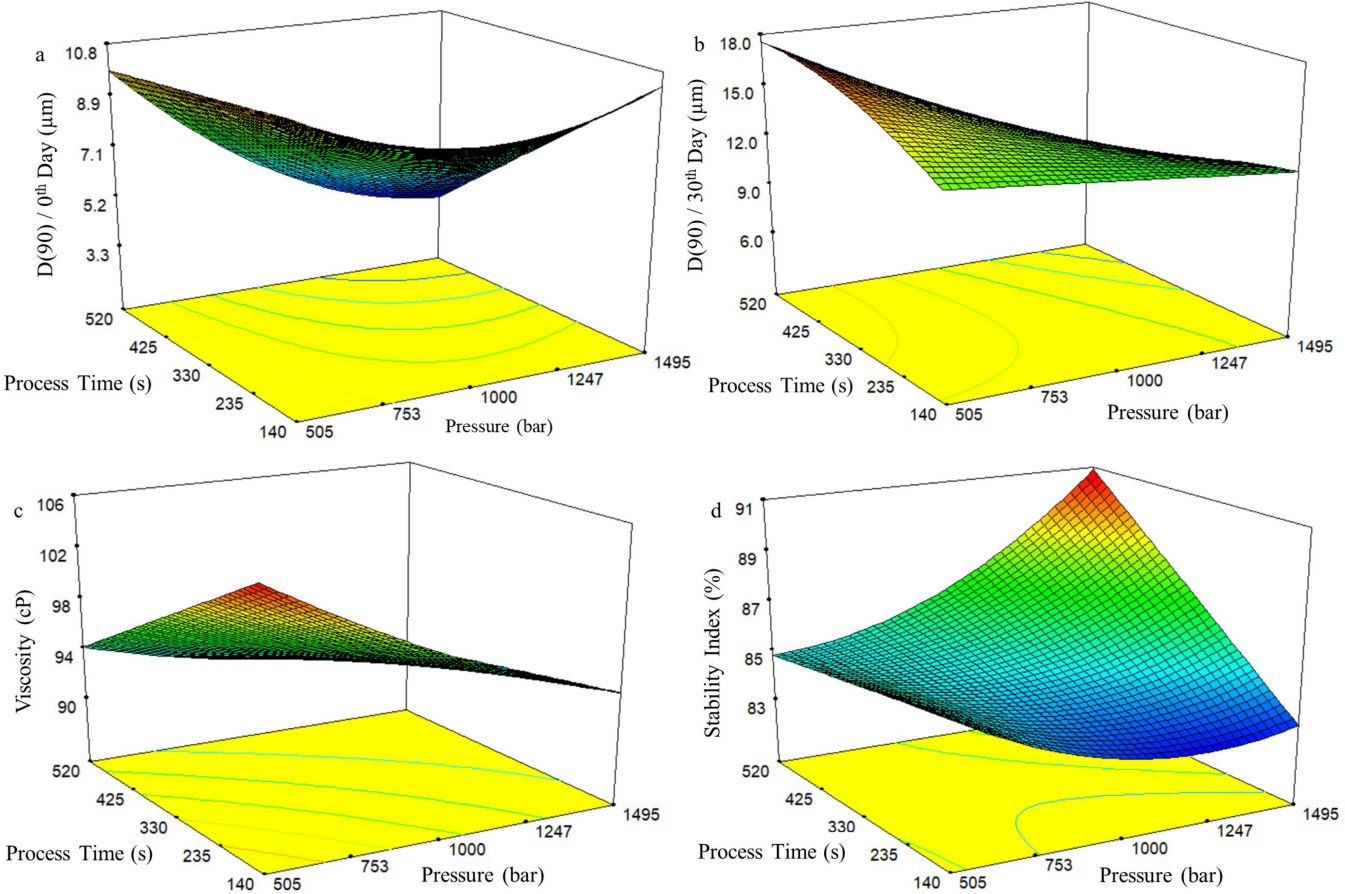
Response surface and contour plots for (a) D(90) at 0th day of storage, (b) D(90) at 30th day of storage, (c) viscosity, and (d) stability index of water‐in‐oil (W/O) emulsions produced with high‐pressure homogenizer.

To visually assess the interaction effect of process variables on responses using the developed models, response surface graphs and contour plots were generated. However, these curves can provide meaningful and realistic insights only if the interaction effects of factors on responses are significant. In the present study, these interaction effects demonstrated significance in 10 out of the 12 derived models, thereby prompting the creation of response surface plots that illustrate these interactions (Figures 1, 2, 3).

For the rotor–stator homogenizer, the interaction effects of factors on D(90) values (both at 0 and 30 days), viscosity, and stability index are depicted in Figure 1a–d. The droplet size decreased as the homogenization intensity increased, evident in both fresh emulsions and those stored for 30 days (Figure 1a,b). This phenomenon aligns with the principle that higher energy in the emulsification process leads to smaller droplets (Bamba et al. 2018; Khalid et al. 2013; Yang et al. 2021). Viscosity and stability index values initially increased and later decreased as the homogenization intensity increased (Figure 1c,d). Santos, Calero, and Muñoz (2016) also noted a similar viscosity trend. The rise in viscosity could be linked to decreased droplet size, enhancing stability. The subsequent viscosity decline (while droplet size remained unchanged) might indicate the breakdown of flocculated emulsion structures. As droplet size reduces, closer spacing between droplets leads to higher viscosity due to hydrodynamic interactions. Moreover, the increased surface area of smaller droplets heightens the importance of the emulsifier layer adsorbed on the surface in relation to droplet size. However, smaller droplets can also experience flocculation. Emulsions with flocculated droplets exhibit higher viscosities, as flocs retain continuous phase, resulting in an effective volume fraction surpassing individual droplet volume fraction (McClements 2016). Elevated emulsification intensity may disrupt flocs, lowering both viscosity and stability.

In ultrasonic homogenization, the effect of sonication amplitude and processing time on the emulsification process was assessed. Statistically significant interaction effects were observed only for viscosity and D(90) value on the 30th day. Longer processing times were expected to elevate viscosity, while increased amplitude with prolonged sonication times was awaited to have an opposite effect on viscosity. As shown in Figure 2b, smaller droplets (D(90) value on the 30th day) were generated at lower amplitude and shorter processing times. This value increased with longer processing times and higher amplitudes. Regarding the interaction between these factors, the droplet size initially increased to a certain point and then decreased slightly. The increase in droplet size might relate to “over‐processing,” characterized by enlargement of droplets and coalescence due to excessive mechanical action. Ultrasonic forces, including primary and secondary Bjerknes forces, are intensified by transmitted energy. Secondary Bjerknes force draws droplets to acoustic field nodes and antinodes, leading to aggregation and “over‐processing” (Kentish et al. 2008). Moreover, higher energy might generate more bubbles, shielding the solution from ultrasonic energy and reducing power transmission (Kentish et al. 2008). This bubble‐induced shielding could also contribute to local over‐processing. Similarly, Kentish et al. (2008) reported larger droplets under high‐energy levels and Hassanshahi et al. (Hassanshahi et al. 2023) also noted that excessive sonication negatively affected emulsion stability.

Response surface graphs for high‐pressure homogenizer–produced samples are illustrated (Figure 3). Figure 3a,b,d indicates that heightened mechanical impact decreased D(90) values while increasing stability index. Droplet‐size reduction associated with higher pressure and prolonged processing time is evident in the literature (Qian and McClements 2011; Tcholakova et al. 2003). Figure 3c shows viscosity reduction with increased pressure and processing time, potentially linked to floc disruption by amplified energy transmission (McClements 2016).

### 3.2 Optimization and Comparison of Emulsification Techniques

The optimization for each homogenizer was performed based on the criteria to obtain the maximum stability index with the minimum viscosity and D(90) values. The significance level and weight of all responses were considered equal. The optimum point was decided with the “desirability function” method for each emulsification technique using the models presented in Table 2 and the obtained optimum conditions are shown in Table 3. The desirability values for the obtained optimum conditions varied from 0.641 to 0.974 (Table 3).

**TABLE 3:** Results of statistical analysis for verification of optimization and technique for order preference by similarity to ideal solution (TOPSIS) analysis.*

Unit	Optimum conditions		Optimization verification							TOPSIS
	Factor	Value	Response	Predicted values	Experimental values	SE	Difference	%Error	*p*	Relative closeness
Rotor–stator	Agitation speed (rpm)	17,800	Viscosity (cP)	94.08	93.13 ± 0.62	0.36	−0:96	1.03	0.117	0.9812
	Time (s)	530	*D*(90) / 0th day (μm)	0.77	0.67 ± 0.05	0.03	−0.10	14.61	0.083	
	Desirability	0.641	Stability index (%)	91.44	93.77 ± 2.07	1.19	2.33	2.48	0.189	
			*D*(90) / 30th day (μm)	0,75	0.77 ± 0.04	0.02	0.02	3.17	0.347	
Ultrasonic homogenizer	Amplitude (%)	39	Viscosity (cP)	97.10	97.52 ± 0.25	0.14	0.41	0.42	0.100	0.1003
	Time (s)	139 s	*D*(90) / 0th day (μm)	4.08	4.07 ± 0.37	0.21	−0.01	0.36	0.955	
	Desirability	0.785	Stability index (%)	89.29	89.57 ± 1.10	0.64	0.28	0.31	0.703	
			*D*(90) / 30th day (μm)	6.09	5.97 ± 0.67	0.38	−0.12	1.95	0.790	
High‐pressure homogenizer	Pressure (bar)	1475	Viscosity (cP)	90.33	90.29 ± 2.06	1.19	−0.04	0.04	0.977	0.1102
	Time (s)	520 s	*D*(90) / 0th day (μm)	3.37	3.55 ± 0.27	0.15	0.18	4.99	0.370	
	Desirability	0.974	Stability index (%)	90.55	90.08 ± 1.10	0.63	−0.47	0.52	0.538	
			*D*(90) / 30th day (μm)	6.93	6.83 ± 1.21	0.70	−0.10	1.41	0.903	

Abbreviation: SE, mean standard error.

* Values show mean ± standard deviation of the analysis results.

In order to validate the theoretical optimum conditions, additional three emulsions were produced at the optimum conditions for each emulsification technique and analyzed. As can be seen in Table 3, the predicted emulsion properties were successfully achieved using all emulsification techniques. Moreover, the *t‐*test results indicated that there were no statistically significant differences between the theoretical and experimental values (*p* > 0.05).

The emulsions, produced under the verified optimal process conditions for each emulsification technique, were subjected to analysis, and the results are detailed in Table 4. Across all three homogenizers, the emulsions exhibited flow behaviors approximating Newtonian, with n values ranging from 0.905 to 0.958 (Table 4). Among the three techniques, ultrasonic homogenization led to the highest viscosity, whereas high‐pressure homogenization resulted in the lowest viscosity (*p* < 0.05). Notably, the rotor–stator homogenizer stood out by yielding emulsions with superior stability indices compared to the others (*p* < 0.05). Similar to these findings, a previous research by Altuntas, Sumnu, and Sahin (2017) also revealed that rotor–stator–produced double emulsions exhibited the highest stability, followed by those from high‐pressure and ultrasonic homogenization.

**TABLE 4:** The physical properties of emulsions produced under optimum processing conditions of three different emulsification techniques.*

Storage	Properties	Rotor–stator	Ultrasonic homogenizer	High‐pressure homogenizer
0th Day	Viscosity (cP)	93.13 ± 0.62^b^	97.52 ± 0.25^c^	90.29 ± 2.06^a^
	*n* (−)	0.958 ± 0.004^b^	0.944 ± 0.001^b^	0.895 ± 0.016^a^
	*K* (Pa.s^n^)	1.064 ± 0.024^a^	1.179 ± 0.001^b^	1.316 ± 0.073^c^
	*D*(90) (μm)	0.67 ± 0.05^a^	4.07 ± 0.37^b^	3.55 ± 0.27^b^
	*D* [2, 3] (μm)	0.423 ± 0.030^a^	0.982 ± 0.540^b^	1.438 ± 0.198^c^
	*D* [3, 4] (μm)	0.479 ± 0.029^a^	1.174 ± 0.097^b^	2.157 ± 0.220^c^
	Span (−)	0.830 ± 0.068^a^	3.168 ± 0.409^b^	1.091 ± 0.01^a^
30th Day	Stability Index (%)	93.77 ± 2.07^b^	89.57 ± 1.10^a^	90.08 ± 1.10^a^
	*D*(90) (μm)	0.77 ± 0.04^a^	5.97 ± 0.67^b^	6.83 ± 1.21^b^
	*D* [2, 3] (μm)	0.485 ± 0.015^a^	1.062 ± 0.220^b^	2.153 ± 0.306^c^
	*D* [3, 4] (μm)	0.583 ± 0.051^a^	2.329 ± 0.646^b^	3.874 ± 0.586^c^
	Span (−)	0.850 ± 0.041^a^	4.190 ± 0.735^b^	1.512 ± 0.094^a^

* Values show mean ± standard deviation of the analysis results and the same superscript letters (a–c) indicate no significant difference between the samples (*p* > 0.05).

Abbreviations: *D*, droplet diameter; *K*, consistency coefficient; *n*, dimensionless flow behavior index.

Emulsions produced using rotor–stator had significantly lower droplet sizes both initially and after 30 days of storage, followed by the emulsions produced with ultrasonic homogenizer (*p* < 0.05) (Table 4). Rotor–stator and high‐pressure homogenized emulsions exhibited comparable narrow droplet‐size distributions, as indicated by similar Span values (*p* > 0.05). Particularly, emulsions derived from the rotor–stator technique presented robust stability and maintained their droplet‐size distribution during storage (Table 4). Although the high‐pressure homogenizer also achieved a narrow droplet‐size distribution based on the Span value, it could not sufficiently minimize the droplet size (Table 4). The high‐pressure homogenization process might lead to increased droplet size if the emulsifier is not able to stabilize the formed droplet area rapidly, thereby impacting the emulsion structure through adsorption kinetics (Neumann et al. 2018).

Table 4 demonstrates that ultrasonic homogenization could produce droplets at 1‐μm scale, but resulting in a distinctively polydisperse droplet‐size distribution. The ability of ultrasound to generate small droplet sizes must be accompanied by enhancements to achieve a homogeneous size distribution. Leong et al. (2017) also observed a bimodal droplet‐size distribution through ultrasonic homogenization, attributing it to simultaneous collisions during size reduction due to strong shear forces within the system. Furthermore, this polydisperse nature could arise from the non‐uniform distribution of ultrasonic power at various points (Kabakci et al. 2021). Kentish et al. (2008) noted that a portion of the fluid flows around the probe without significant interaction or only minimal interaction with the “hot zone” at the probe's tip, which might elucidate the emergence of a polydisperse structure during continuous flow ultrasonic homogenization.

The predicted suitable emulsification technique for producing the primary W/O emulsion with the highest stability and lowest viscosity was determined by employing TOPSIS with the predefined optimization targets (based on the responses such as viscosity, stability index, and D(90) values immediately after emulsion preparation and following 30 days of storage) at equal weights. The calculated “relative closeness” values ranged from 0.1003 to 0.9812, with the rotor–stator–produced emulsion standing out by achieving a relative closeness value nearly approaching 1 (Table 3). In a previous study conducted by the authors, the effects of the formulation of W/O emulsions loaded with casein hydrolysate were investigated and the most appropriate formulation, which was used in the present study, was determined (Salum, Ulubaş, et al. 2023). The optimal W/O emulsion in the previous study had a viscosity of 139.8 cP and a D[4,3] value of 0.662 μm (Salum, Ulubaş, et al. 2023). However, for the emulsions produced using RS with the same formulation in the present study, the viscosity and D[4,3] value were found to be 93.1 cP and 0.479 μm, respectively (Table 4). This variation indicates that both the optimization of the homogenization process and the formulation have a significant impact on the emulsion properties.

### 3.3 Comparison of W_1_ /O/W_2_ Emulsions

Double emulsions were created to assess the suitability of primary emulsions for double emulsion production using primary emulsions produced under optimal conditions. Measurements were conducted on the retention efficiency of the internal phase, stability, droplet size, and viscosity values of the W_1_/O/W_2_ emulsions. Additionally, the retention efficiency of the internal phase and droplet‐size distributions of double emulsions were evaluated after 8 days of storage at room conditions to gauge the stability of the double emulsions (Table 5).

**TABLE 5:** The physical properties of double emulsions*.

Homogenizer/storage time (day)	REI (%)	*D*(90) (μm)	*D*[3,2] (μm)	*D*[4,3] (μm)	Span (−)	Viscosity (cP)	*n* (−)	*K* (Pa.s^n^)
RS/0	82.4 ± 0.25^a^	9.77 ± 0.35^c^	3.63 ± 0.19^c^	5.52 ± 0.25^c^	1.53 ± 0.04^a^	6.93 ± 0.08^c^	1.024 ± 0.013^a^	0.061 ± 0.005^b^
US/0	82.1 ± 0.51^a^	8.85 ± 0.17^b^	2.98 ± 0.05^b^	4.82 ± 0.07^b^	1.69 ± 0.01^b^	3.75 ± 0.08^b^	1.015 ± 0.010^a^	0.035 ± 0.002^a^
HP/0	82.4 ± 0.36^a^	7.89 ± 0.06^a^	2.42 ± 0.04^a^	3.97 ± 0.10^a^	2.03 ± 0.01^c^	3.18 ± 0.04^a^	1.028 ± 0.011^a^	0.027 ± 0.001^a^
RS/8	74.3 ± 0.07^c+^	10.30 ± 0.20^c^	3.65 ± 0.10^c^	5.66 ± 0.13^c^	1.62 ± 0.05^a^	—	—	—
US/8	70.2 ± 0.04^b+^	8.84 ± 0.11^b^	2.89 ± 0.09^b^	4.83 ± 0.14^b^	1.84 ± 0.03^b+^	—	—	—
HP/8	69.0 ± 0.36^a+^	7.75 ± 0.14^a^	2.41 ± 0.04^a^	3.91 ± 0.06^a^	2.05 ± 0.03^c^	—	—	—

Abbreviations: *D*, droplet diameter; HP, high‐pressure homogenizer; *K*, consistency coefficient; *n*, dimensionless flow behavior index; REI, the retention efficiency of the internal phase; RS, rotor–stator homogenizer; US, ultrasonic homogenizer.

* Values show mean ± standard deviation of the analysis results and the same superscript letters (a–c) indicate no significant difference between the samples (*p* > 0.05). + (as superscript) indicates significant difference between the 0th and 8th day samples according to *t*‐test (*p* < 0.05).

To begin with, a key distinction between W_1_/O and W_1_/O/W_2_ emulsions is that W_1_/O exhibit smaller droplet sizes and higher viscosity (Tables 4 and 5). The reduced droplet size in W_1_/O is attributed to the application of high mechanical force during their preparation (Lee et al. 2013). In contrast, W_1_/O/W_2_ emulsions are prepared under milder conditions to minimize damage to the W_1_/O. In addition, the disparity in viscosity arises primarily because W_1_/O had higher oil content than W_1_/O/W_2_ emulsions and the viscosity of the oil phase in the present study is higher than that of the water phase. Furthermore, W_1_/O had the smaller droplet sizes that contribute to their higher viscosity (Pal 1996).

**FIGURE 4:**
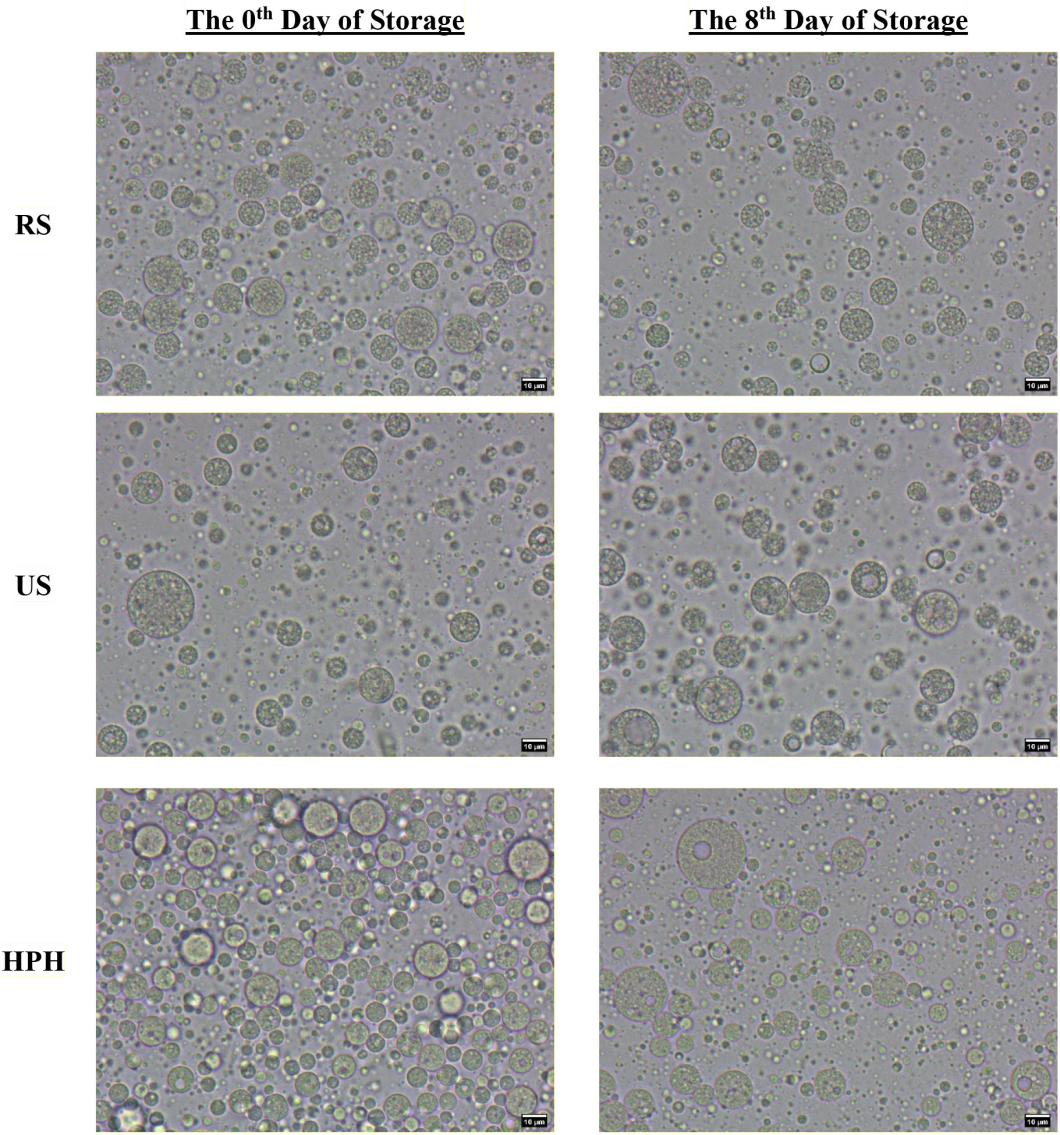
Microscope images of double emulsions on the 0th and 8th days of storage.

For W_1_/O/W_2_, no statistically significant difference was observed between the retention efficiency of the internal phase in fresh emulsions produced with different primary emulsions. However, this scenario changed significantly on the 8th day of storage (*p* > 0.05). While the retention efficiencies of the internal phase significantly decreased for all double emulsions after 8 days of storage, the highest values were found in the double emulsions prepared with the primary emulsion produced using RS, followed by US. Altuntas, Sumnu, and Sahin (2017) noted that double emulsions containing primary emulsions produced with RS were more stable compared to those produced with US and a microfluidizer. In this context, it is hypothesized that the decrease in retention efficiency of the internal phase may be attributed to the diffusion of the internal phase. Microscope images revealed growth in the internal water droplets due to coalescence on the 8th day of storage (Figure 4). As emphasized in the previous section, the droplet sizes of primary emulsions produced with RS were small and uniform compared to other emulsions, which may explain its higher retention efficiency of the internal phase than others.

Although the largest droplet sizes were detected in the double emulsions prepared with RS primary emulsion, the Span value of this sample was lower than those of the others. Additionally, the viscosity of this sample was higher than those of other samples, which may contribute to lower creaminess in comparison. While reducing droplet size might lead to increased viscosity, a decrease in polydispersity is also expected to raise viscosity (Pal 1996). Therefore, the higher viscosity observed in RS samples may be associated with their narrower droplet‐size distribution.

**FIGURE 5:**
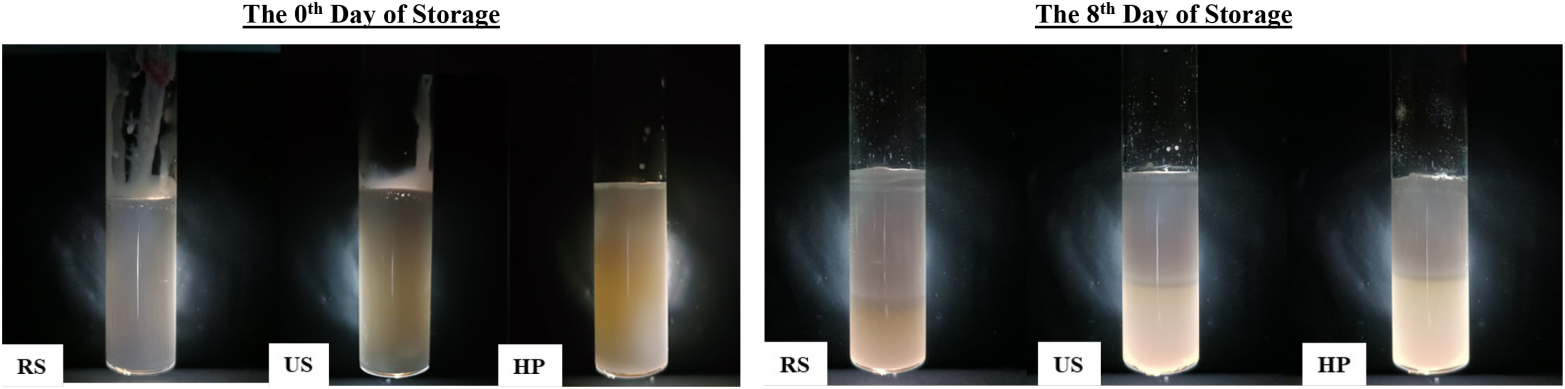
Creaming photographs of double emulsions on the 0th and 8th days of storage.

Creaming in the double emulsions was not visually evident after 8 days of storage. To make potential creaming visually detectable, the double emulsions were examined in a dark environment with LED light from behind, and the images obtained under this condition are depicted in Figure 5. According to Stokes' theorem, as the viscosity of a fluid increases, the velocity of the droplets dispersed in this fluid decreases, and this velocity is directly proportional to the droplet diameter (D) (Yildirim, Sumnu, and Sahin 2017). Thus, it is inferred that higher viscosity and smaller droplet sizes impede creaming. Additionally, double emulsions including primary emulsions prepared with RS exhibit greater viscosity and narrower droplet‐size distribution compared to other samples, despite having larger droplet sizes.

## 4 Conclusions

This study aimed to produce W/O emulsions as the dispersed phase for casein hydrolysate–loaded double emulsions. To achieve this, three commonly used homogenization techniques for primary emulsions were individually optimized, and their effectiveness at the identified optimum points was compared.

In accordance with common expectations, the augmentation of homogenization intensity caused a reduction in droplet size, which corresponded to increased viscosity and enhanced stability. However, the conventional pattern observed was disrupted in this study, with instances of over‐processing being identified in both the rotor–stator and ultrasonic homogenizers under specific experimental conditions. Notably, the ultrasonic homogenizer exhibited its smallest droplet size when subjected to the lowest energy transmission.

The optimum conditions for each technique were as follows: 530 s at 17,800 rpm agitation speed for the rotor–stator homogenizer, 139 s at 39% amplitude for the ultrasonic homogenizer, and 520 s at 1475 bar for the high‐pressure homogenizer. Among these, the most suitable primary emulsion was obtained through the rotor–stator homogenizer, characterized by viscosity, stability index, D(90) value for the initial emulsion, and D(90) value after 30 days of storage, measuring 93.1 cP, 93.8%, 0.67, and 0.75 μm, respectively.

The present study showed that one can utilize primary emulsions with identical formulations generated through diverse homogenization techniques to achieve varied properties in double emulsions. To optimize the retention efficiency of the internal phase, it is advantageous to minimize the droplet size of the internal water phase in the primary emulsion and maintain a monodisperse distribution. In contrast, for double emulsions, a relatively larger droplet size in the primary emulsion with a monodisperse distribution proves beneficial.

## Author Contributions

**Pelin Salum:** conceptualization (equal), data curation (equal), formal analysis (equal), investigation (equal), visualization (equal), writing – original draft (equal). **Çağla Ulubaş:** data curation (equal), formal analysis (equal). **Onur Güven:** data curation (equal), formal analysis (equal), methodology (equal). **Mustafa Cam:** conceptualization (equal), methodology (equal), resources (equal), writing – review and editing (equal). **Levent Yurdaer Aydemir:** methodology (equal), resources (equal), supervision (equal), writing – review and editing (equal). **Zafer Erbay:** conceptualization (equal), data curation (equal), funding acquisition (equal), methodology (equal), project administration (equal), resources (equal), writing – review and editing (equal).

## Ethics Statement

This article does not contain any studies with human or animal subjects.

## Conflicts of Interest

The authors declare no conflicts of interest.

## Supporting information


Data S1.

## Data Availability

The datasets generated during and/or analyzed during the current study are available from the corresponding author on reasonable request.

## References

[fsn34525-bib-0001] Aguilar‐Toala, J. E. , D. Quintanar‐Guerrero , A. M. Liceaga , and M. L. Zambrano‐Zaragoza . 2022. “Encapsulation of Bioactive Peptides: A Strategy to Improve the Stability, Protect the Nutraceutical Bioactivity and Support Their Food Applications.” RSC Advances 12: 6449–6458. 10.1039/d1ra08590e. 35424621 PMC8982217

[fsn34525-bib-0002] Altuntas, O. Y. , G. Sumnu , and S. Sahin . 2017. “Preparation and Characterization of W/O/W Type Double Emulsion Containing PGPR–Lecithin Mixture as Lipophilic Surfactant.” Journal of Dispersion Science and Technology 38: 486–493. 10.1080/01932691.2016.1179121.

[fsn34525-bib-0003] Azarikia, F. , S. Abbasi , M. G. Scanlon , and D. J. McClements . 2017. “Emulsion Stability Enhancement Against Environmental Stresses Using Whey Protein–Tragacanthin Complex: Comparison of Layer‐By‐Layer and Mixing Methods.” International Journal of Food Properties 20: 2084–2095. 10.1080/10942912.2017.1362651.

[fsn34525-bib-0004] Bamba, B. S. B. , J. Shi , C. C. Tranchant , et al. 2018. “Coencapsulation of Polyphenols and Anthocyanins From Blueberry Pomace by Double Emulsion Stabilized by Whey Proteins: Effect of Homogenization Parameters.” Molecules 23: 2525. 10.3390/molecules23102525. 30279378 PMC6222392

[fsn34525-bib-0005] Chevalier, R. C. , A. Gomes , and R. L. Cunha . 2021. “Tailoring W/O Emulsions for Application as Inner Phase of W/O/W Emulsions: Modulation of the Aqueous Phase Composition.” Journal of Food Engineering 297: 110482. 10.1016/j.jfoodeng.2021.110482.

[fsn34525-bib-0006] Erbay, Z. , and F. Icier . 2009. “Optimization of Hot Air Drying of Olive Leaves Using Response Surface Methodology.” Journal of Food Engineering 91: 533–541.

[fsn34525-bib-0007] Giroldi, M. , I. M. Grambusch , D. N. Lehn , and C. F. V. de Souza . 2021. “Encapsulation of Dairy Protein Hydrolysates: Recent Trends and Future Prospects.” Drying Technology 39: 1513–1528. 10.1080/07373937.2021.1906695.

[fsn34525-bib-0008] Hassanshahi, N. , G. Hu , K. Lee , and J. Li . 2023. “Effect of Ultrasonic Homogenization on Crude Oil‐Water Emulsion Stability.” Journal of Environmental Science and Health ‐ Part A 58: 211–221. 10.1080/10934529.2023.2178788. 36803402

[fsn34525-bib-0009] Heidari, F. , S. M. Jafari , A. M. Ziaiifar , and N. Malekjani . 2022. “Stability and Release Mechanisms of Double Emulsions Loaded With Bioactive Compounds; a Critical Review.” Advances in Colloid and Interface Science 299: 102567. 10.1016/j.cis.2021.102567. 34839180

[fsn34525-bib-0010] Himmetagaoglu, A. B. , Z. Erbay , and M. Cam . 2018. “Production of Microencapsulated Cream: Impact of Wall Materials and Their Ratio.” International Dairy Journal 83: 20–27. 10.1016/j.idairyj.2018.03.007.

[fsn34525-bib-0011] Hwang, C. L. , and K. Yoon . 1981. Multiple Attribute Decision Making: Methods and Applications. New York, USA: Springer.

[fsn34525-bib-0012] Jo, Y. J. , H. P. Karbstein , and U. S. Van Der Schaaf . 2019. “Collagen Peptide‐Loaded W1/O Single Emulsions and W1/O/W2 Double Emulsions: Influence of Collagen Peptide and Salt Concentration, Dispersed Phase Fraction and Type of Hydrophilic Emulsifier on Droplet Stability and Encapsulation Efficiency.” Food & Function 10: 3312–3323. 10.1039/c8fo02467g. 31095142

[fsn34525-bib-0013] Kabakci, C. , G. Sumnu , S. Sahin , and M. H. Oztop . 2021. “Encapsulation of Magnesium With Lentil Flour by Using Double Emulsion to Produce Magnesium Enriched Cakes.” Food and Bioprocess Technology 14: 1773–1790. 10.1007/s11947-021-02672-5.

[fsn34525-bib-0014] Kentish, S. , T. J. Wooster , M. Ashokkumar , S. Balachandran , R. Mawson , and L. Simons . 2008. “The Use of Ultrasonics for Nanoemulsion Preparation.” Innovative Food Science and Emerging Technologies 9: 170–175. 10.1016/j.ifset.2007.07.005.

[fsn34525-bib-0015] Khalid, N. , I. Kobayashi , M. A. Neves , K. Uemura , and M. Nakajima . 2013. “Preparation and Characterization of Water‐In‐Oil Emulsions Loaded With High Concentration of L‐Ascorbic Acid.” LWT‐ Food Science and Technology 51: 448–454. 10.1016/j.lwt.2012.11.020. 23748753

[fsn34525-bib-0016] Kim, J. W. , H. H. Kang , S. K. Do , and S. G. Oh . 2003. “Stabilization of Water‐Soluble Antioxidant in Water‐In‐Oil‐In‐Water Double Emulsions.” Journal of Dispersion Science and Technology 24: 833–839. 10.1081/DIS-120025551.

[fsn34525-bib-0017] Klojdová, I. , J. Štětina , and Š. Horáčková . 2019. “W/O/W Multiple Emulsions as the Functional Component of Dairy Products.” Chemical Engineering and Technology 42: 715–727. 10.1002/ceat.201800586.

[fsn34525-bib-0018] Korhonen, H. 2002. “Technology Options for New Nutritional Concepts.” International Journal of Dairy Technology 55: 79–88. 10.1046/j.1471-0307.2002.00050.x.

[fsn34525-bib-0019] Kumar, A. , R. Kaur , V. Kumar , S. Kumar , R. Gehlot , and P. Aggarwal . 2022. “New Insights Into Water‐In‐Oil‐In‐Water (W/O/W) Double Emulsions: Properties, Fabrication, Instability Mechanism, and Food Applications.” Trends in Food Science and Technology 128: 22–37. 10.1016/j.tifs.2022.07.016.

[fsn34525-bib-0020] Lee, L. L. , N. Niknafs , R. D. Hancocks , and I. T. Norton . 2013. “Emulsification: Mechanistic Understanding.” Trends in Food Science and Technology 31: 72–78. 10.1016/j.tifs.2012.08.006.

[fsn34525-bib-0021] Leong, T. S. H. , M. Zhou , N. Kukan , M. Ashokkumar , and G. J. O. Martin . 2017. “Preparation of Water‐In‐Oil‐In‐Water Emulsions by Low Frequency Ultrasound Using Skim Milk and Sunflower Oil.” Food Hydrocolloids 63: 685–695. 10.1016/j.foodhyd.2016.10.017.

[fsn34525-bib-0022] McClements, D. J. 2016. Food Emulsions: Principles, Practice and Techniques. 3rd ed. Broken Sound Parkway NW: CRC Press.

[fsn34525-bib-0023] Muschiolik, G. , and E. Dickinson . 2017. “Double Emulsions Relevant to Food Systems: Preparation, Stability, and Applications.” Comprehensive Reviews in Food Science and Food Safety 16: 532–555. 10.1111/1541-4337.12261. 33371556

[fsn34525-bib-0024] Myers, R. H. , and D. C. Montgomery . 2002. Response Surface Methodology: Process and Product Optimization Using Designed Experiments. 2nd ed. New York: John Wiley and Sons.

[fsn34525-bib-0025] Neumann, S. M. , N. Wittstock , U. S. van der Schaaf , and H. P. Karbstein . 2018. “Interactions in Water in Oil in Water Double Emulsions: Systematical Investigations on the Interfacial Properties and Emulsion Structure of the Outer Oil in Water Emulsion.” Colloids and Surfaces A: Physicochemical and Engineering Aspects 537: 524–531. 10.1016/j.colsurfa.2017.10.070.

[fsn34525-bib-0026] Pal, R. 1996. “Effect of Droplet Size on the Rheology of Emulsions.” AICHE Journal 42: 3181–3190. 10.1002/aic.690421119.

[fsn34525-bib-0027] Paula, D. D. A. , E. B. de Oliveira , A. V. N. de Carvalho Teixeira , A. D. S. Soares , and A. M. Ramos . 2018. “Double Emulsions (W/O/W): Physical Characteristics and Perceived Intensity of Salty Taste.” International Journal of Food Science and Technology 53: 475–483. 10.1111/ijfs.13606.

[fsn34525-bib-0028] Qian, C. , and D. J. McClements . 2011. “Formation of Nanoemulsions Stabilized by Model Food‐Grade Emulsifiers Using High‐Pressure Homogenization: Factors Affecting Particle Size.” Food Hydrocolloids 25: 1000–1008. 10.1016/j.foodhyd.2010.09.017.

[fsn34525-bib-0029] Raviadaran, R. , M. H. Ng , S. Manickam , and D. Chandran . 2019. “Ultrasound‐Assisted Water‐In‐Palm Oil Nano‐Emulsion: Influence of Polyglycerol Polyricinoleate and NaCl on its Stability.” Ultrasonics Sonochemistry 52: 353–363. 10.1016/j.ultsonch.2018.12.012. 30555038

[fsn34525-bib-0030] Salum, P. , S. Berktas , D. Bas , M. Cam , and Z. Erbay . 2023. “Optimization of Spray Drying Conditions for Improved Physical Properties in the Production of Enzyme‐Modified Cheese Powder.” Journal of Food Science 88: 244–258. 10.1111/1750-3841.16392. 36463415

[fsn34525-bib-0031] Salum, P. , S. Berktas , P. Kendirci , D. Bas , M. Cam , and Z. Erbay . 2023. “Production of Microencapsulated Enzyme‐Modified Cheese (EMC) Powder: Impact of Wall Material Combinations, Their Concentrations, and Homogenisation Pressures.” International Dairy Journal 143: 105687. 10.1016/j.idairyj.2023.105687.

[fsn34525-bib-0032] Salum, P. , O. Güven , L. Y. Aydemir , and Z. Erbay . 2022. “Microscopy‐Assisted Digital Image Analysis With Trainable Weka Segmentation (TWS) for Emulsion Droplet Size Determination.” Coatings 12: 364. 10.3390/coatings12030364.

[fsn34525-bib-0033] Salum, P. , Ç. Ulubaş , O. Güven , L. Y. Aydemir , and Z. Erbay . 2023. “Casein‐Hydrolysate‐Loaded W/O Emulsion Preparation as the Primary Emulsion of Double Emulsions: Effects of Varied Phase Fractions, Emulsifier Types, and Concentrations.” Colloids and Interfaces 7, no. 1: 1–14.

[fsn34525-bib-0034] Salum, P. , Ç. Ulubaş , O. Güven , M. Cam , L. Y. Aydemir , and Z. Erbay . 2024. “Design and Process Optimisation of Double Emulsions Loaded With Casein Hydrolysate.” International Dairy Journal 157: 106026. 10.1016/j.idairyj.2024.106026.

[fsn34525-bib-0035] Santos, J. , N. Calero , and J. Muñoz . 2016. “Optimization of a Green Emulsion Stability by Tuning Homogenization Rate.” RSC Advances 6: 57563–57568. 10.1039/c6ra10207g.

[fsn34525-bib-0036] Taha, A. , E. Ahmed , A. Ismaiel , et al. 2020. “Ultrasonic Emulsification: An Overview on the Preparation of Different Emulsifiers‐Stabilized Emulsions.” Trends in Food Science and Technology 105: 363–377. 10.1016/j.tifs.2020.09.024.

[fsn34525-bib-0037] Tcholakova, S. , N. D. Denkov , D. Sidzhakova , I. B. Ivanov , and B. Campbell . 2003. “Interrelation Between Drop Size and Protein Adsoption at Various Emulsification Conditions.” Langmuir 19: 5640–5649.

[fsn34525-bib-0038] Ushikubo, F. Y. , and R. L. Cunha . 2014. “Stability Mechanisms of Liquid Water‐In‐Oil Emulsions.” Food Hydrocolloids 34: 145–153. 10.1016/j.foodhyd.2012.11.016.

[fsn34525-bib-0039] van der Graaf, S. , C. G. P. H. Schroën , and R. M. Boom . 2005. “Preparation of Double Emulsions by Membrane Emulsification ‐ A Review.” Journal of Membrane Science 251: 7–15. 10.1016/j.memsci.2004.12.013.

[fsn34525-bib-0040] Yang, W. , J. Li , D. Ren , et al. 2021. “Construction of a Water‐In‐Oil‐In‐Water (W/O/W) Double Emulsion System Based on Oyster Peptides and Characterisation of Freeze‐Dried Products.” International Journal of Food Science and Technology 56: 6635–6648. 10.1111/ijfs.15354.

[fsn34525-bib-0041] Ye, M. , S. Kim , and K. Park . 2010. “Issues in Long‐Term Protein Delivery Using Biodegradable Microparticles.” Journal of Controlled Release 146: 241–260. 10.1016/j.jconrel.2010.05.011. 20493221

[fsn34525-bib-0042] Yildirim, M. , G. Sumnu , and S. Sahin . 2017. “The Effects of Emulsifier Type, Phase Ratio, and Homogenization Methods on Stability of the Double Emulsion.” Journal of Dispersion Science and Technology 38: 807–814. 10.1080/01932691.2016.1201768.

