# Supplementary material for: The Impact of Homogenization Techniques and Conditions on Water‐In‐Oil Emulsions for Casein Hydrolysate–Loaded Double Emulsions: A Comparative Study
Source: Food Sci Nutr. 2024 Oct 18;12(11):9585–99. doi: 10.1002/fsn3.4525 (PMC11606908; doi:10.1002/fsn3.4525)
Supplement: Supplementary file 1 — Data S1. [file FSN3-12-9585-s001.docx]

**SUPPLEMENTARY FILE**

**The Impact of Homogenization Techniques and Conditions on Water-in-Oil Emulsions for Casein Hydrolysate-Loaded Double Emulsions: A Comparative Study**

Pelin Salum^1^, Çağla Ulubaş^1^, Onur Güven^2^, Mustafa Cam^3^, Levent Yurdaer Aydemir^1^, Zafer Erbay^1,*^

*^1^ Department of Food Engineering, Faculty of Engineering, Adana Alparslan Turkes Science and Technology University, 01250 Adana, Turkey.*

*^2^ Department of Mining Engineering, Faculty of Engineering, Adana Alparslan Turkes Science and Technology University, 01250 Adana, Turkey.*

*^3^ Department of Food Engineering, Faculty of Engineering, Erciyes University, 38039 Kayseri, Turkey.*

*Corresponding author:

Mailing address: Department of Food Engineering, Faculty of Engineering, Adana Alparslan Turkes Science and Technology University, 01250, Saricam, Adana, Turkey, Tel: +90 322 4550000 (2080), Fax: +90 322 4550009

E-mail: [zafererbay@yahoo.com](mailto:zafererbay@yahoo.com); [zerbay@atu.edu.tr](mailto:zerbay@atu.edu.tr)

**Table S1.** Properties of emulsions produced according to experimental design for rotor-stator homogenizer.^*^

| # | Viscosity  (cP) | n  (-) | K  (Pa.s^n^) | EC  (µS/s) | T  (°C) | 0^th^ Day | | | Stability Index  (%) | 30^th^ Day | | |  |
| --- | --- | --- | --- | --- | --- | --- | --- | --- | --- | --- | --- | --- | --- |
|  |  |  |  |  |  | D(90)  (µm) | D[3,2]  (µm) | D[4,3]  (µm) |  | D(90)  (µm) | D[3,2]  (µm) | D[4,3]  (µm) | |
| RS-1 | 94.5 | 0.93 | 1.19 | 0.02 | 52.8 | 0.75 | 0.41 | 0.47 | 93.10 | 0.71 | 0.45 | 0.55 | |
| RS-2 | 99.8 | 0.95 | 1.19 | 0.02 | 38.1 | 0.98 | 0.48 | 0.59 | 95.91 | 1.13 | 0.72 | 1.00 | |
| RS-3 | 97.0 | 0.94 | 1.19 | 0.02 | 50.1 | 0.65 | 0.39 | 0.44 | 91.74 | 0.67 | 0.43 | 0.51 | |
| RS-4 | 108.3 | 0.94 | 1.32 | 0.02 | 38.9 | 1.34 | 0.50 | 0.67 | 90.81 | 1.28 | 0.76 | 0.88 | |
| RS-5 | 101.0 | 0.94 | 1.22 | 0.02 | 41.2 | 0.93 | 0.45 | 0.56 | 91.92 | 0.77 | 0.50 | 0.56 | |
| RS-6 | 99.1 | 0.95 | 1.18 | 0.02 | 39.1 | 1.14 | 0.51 | 0.65 | 93.22 | 0.86 | 0.56 | 0.62 | |
| RS-7 | 96.9 | 0.95 | 1.16 | 0.02 | 39.3 | 0.96 | 0.62 | 0.70 | 92.69 | 0.95 | 0.63 | 0.71 | |
| RS-8 | 103.5 | 0.95 | 1.22 | 0.02 | 41.7 | 1.21 | 0.69 | 0.81 | 95.38 | 1.19 | 0.73 | 0.83 | |
| RS-9 | 51.2 | 0.56 | 2.50 | 1107 | 26.0 | 10.53 | 2.90 | 4.91 | 60.99 | 13.01 | 3.25 | 5.88 | |
| RS-10 | 97.9 | 0.95 | 1.17 | 0.02 | 38.3 | 1.28 | 0.75 | 0.87 | 88.61 | 1.36 | 0.83 | 0.94 | |
| RS-11 | 47.0 | 0.58 | 2.17 | 1013 | 26.3 | 12.78 | 3.78 | 6.02 | 59.81 | 23.23 | 4.64 | 12.19 | |
| RS-12 | 97.4 | 0.95 | 1.16 | 0.02 | 32.5 | 1.42 | 0.82 | 1.04 | 92.23 | 1.84 | 1.03 | 1.21 | |
| RS-13 | 94.5 | 0.96 | 1.08 | 0.02 | 31.3 | 1.64 | 0.88 | 1.08 | 89.56 | 5.05 | 1.02 | 1.96 | |

**^*^** D, droplet diameter; EC, electrical conductivity; K, consistency coefficient; n, flow behavior index RS, rotor-stator; T, temperature.

**Table S2.** Properties of emulsions produced according to experimental design for ultrasonic homogenizer.^*^

| # | Viscosity  (cP) | n  (-) | K  (Pa.s^n^) | EC  (µS/s) | T  (°C) | 0^th^ Day | | | Stability Index  (%) | 30^th^ Day | | | |
| --- | --- | --- | --- | --- | --- | --- | --- | --- | --- | --- | --- | --- | --- |
|  |  |  |  |  |  | D(90)  (µm) | D[3,2]  (µm) | D[4,3]  (µm) |  | D(90)  (µm) | D[3,2]  (µm) | D[4,3]  (µm) |  |
| US-1 | 101.1 | 0.94 | 1.23 | 0.02 | 68.5 | 5.52 | 1.08 | 2.75 | 83.76 | 13.99 | 6.53 | 10.58 |  |
| US-2 | 97.6 | 0.95 | 1.15 | 0.02 | 43.0 | 3.53 | 0.84 | 1.41 | 89.94 | 5.37 | 1.14 | 2.41 |  |
| US-3 | 102.1 | 0.95 | 1.20 | 0.02 | 66.6 | 5.41 | 1.44 | 2.96 | 83.72 | 13.67 | 7.58 | 9.87 |  |
| US-4 | 103.6 | 0.94 | 1.20 | 0.02 | 58.5 | 3.52 | 1.17 | 1.88 | 84.92 | 6.99 | 2.42 | 4.46 |  |
| US-5 | 93.4 | 0.95 | 1.11 | 0.02 | 34.6 | 6.38 | 1.38 | 2.90 | 92.23 | 13.05 | 2.72 | 6.62 |  |
| US-6 | 103.3 | 0.96 | 1.20 | 0.02 | 69.8 | 5.44 | 1.00 | 2.46 | 85.37 | 12.02 | 4.28 | 7.40 |  |
| US-7 | 101.2 | 0.96 | 1.17 | 0.02 | 69.3 | 5.65 | 1.03 | 2.44 | 84.58 | 12.28 | 5.65 | 9.56 |  |
| US-8 | 100.6 | 0.93 | 1.14 | 0.02 | 64.5 | 4.29 | 1.16 | 2.20 | 83.83 | 12.03 | 5.41 | 8.55 |  |
| US-9 | 98.8 | 0.96 | 1.14 | 0.02 | 80.0 | 9.19 | 1.83 | 4.50 | 82.38 | 15.93 | 6.81 | 11.54 |  |
| US-10 | 96.8 | 0.95 | 1.15 | 0.02 | 85.3 | 8.22 | 2.75 | 5.25 | 83.58 | 11.88 | 4.86 | 7.86 |  |
| US-11 | 103.9 | 0.93 | 1.17 | 0.02 | 70.0 | 5.68 | 1.01 | 2.11 | 85.21 | 12.64 | 5.43 | 8.65 |  |
| US-12 | 101.6 | 0.98 | 1.20 | 0.02 | 72.2 | 6.41 | 1.75 | 3.98 | 84.29 | 12.17 | 5.17 | 8.08 |  |
| US-13 | 95.9 | 0.94 | 1.15 | 0.02 | 48.9 | 6.05 | 0.85 | 2.23 | 89.09 | 15.67 | 3.81 | 10.61 |  |

**^*^** D, droplet diameter; EC, electrical conductivity; K, consistency coefficient; n, flow behavior index; US, ultrasonic homogenizer; T, temperature.

**Table S3.** Properties of emulsions produced according to experimental design for high-pressure homogenizer.^*^

| # | Viscosity  (cP) | n  (-) | K  (Pa.s^n^) | EC  (µS/s) | T  (°C) | 0^th^ Day | | | Stability Index  (%) | 30^th^ Day | | |
| --- | --- | --- | --- | --- | --- | --- | --- | --- | --- | --- | --- | --- |
|  |  |  |  |  |  | D(90)  (µm) | D[3,2]  (µm) | D[4,3]  (µm) |  | D(90)  (µm) | D[3,2]  (µm) | D[4,3]  (µm) |
| HP-1 | 99.2 | 0.92 | 1.30 | 0.02 | 38.2 | 9.91 | 2.61 | 5.51 | 83.41 | 11.98 | 3.16 | 7.43 |
| HP-2 | 96.6 | 0.95 | 1.15 | 0.02 | 50.4 | 7.24 | 3.24 | 4.97 | 86.47 | 13.41 | 5.13 | 8.73 |
| HP-3 | 95.2 | 0.95 | 1.12 | 0.02 | 46.4 | 6.44 | 2.55 | 4.17 | 85.55 | 13.28 | 5.91 | 8.56 |
| HP-4 | 91.7 | 0.94 | 1.12 | 0.02 | 70.4 | 3.82 | 1.24 | 2.04 | 90.40 | 6.93 | 3.09 | 4.76 |
| HP-5 | 90.1 | 0.95 | 1.07 | 0.02 | 64.1 | 7.30 | 3.29 | 4.83 | 89.45 | 8.52 | 3.10 | 4.71 |
| HP-6 | 88.6 | 0.95 | 1.05 | 0.02 | 57.1 | 4.12 | 1.58 | 2.28 | 87.97 | 11.09 | 5.50 | 7.51 |
| HP-7 | 92.0 | 0.96 | 1.06 | 0.02 | 57.4 | 8.17 | 2.22 | 4.04 | 85.02 | 13.87 | 5.93 | 8.40 |
| HP-8 | 94.8 | 0.96 | 1.09 | 0.02 | 48.2 | 10.71 | 2.44 | 5.58 | 84.48 | 17.51 | 6.70 | 11.36 |
| HP-9 | 94.5 | 0.95 | 1.11 | 0.02 | 56.5 | 5.85 | 2.18 | 3.56 | 88.14 | 13.47 | 5.25 | 8.31 |
| HP-10 | 95.3 | 0.94 | 1.17 | 0.02 | 56.5 | 6.97 | 2.29 | 3.88 | 83.84 | 13.01 | 5.16 | 9.00 |
| HP-11 | 94.5 | 0.95 | 1.12 | 0.02 | 57.1 | 10.60 | 2.80 | 6.11 | 83.65 | 12.06 | 4.01 | 7.15 |
| HP-12 | 102.0 | 0.95 | 1.20 | 0.02 | 39.9 | 11.57 | 2.36 | 5.59 | 87.24 | 17.94 | 2.92 | 9.50 |
| HP-13 | 105.4 | 1.01 | 1.03 | 0.02 | 39.5 | 11.43 | 2.05 | 5.12 | 85.89 | 13.67 | 2.30 | 6.65 |

**^*^** D, droplet diameter; EC, electrical conductivity; K, consistency coefficient; n, flow behavior index; HP, high-pressure homogenizer; T, temperature.
